# Beyond the Dual Paraneoplastic Syndromes of Small-Cell Lung Cancer with ADH and ACTH Secretion: A Case Report with Literature Review and Future Implications

**DOI:** 10.1155/2018/4038397

**Published:** 2018-10-18

**Authors:** Krishna Adit Agarwal, Myat Han Soe

**Affiliations:** Department of Medicine, Baystate Medical Center, University of Massachusetts Medical School, Springfield, MA, USA

## Abstract

We present a case of small-cell lung cancer (SCLC) with syndrome of inappropriate antidiuretic hormone secretion (SIADH) in which serum sodium gradually normalized with the onset of hypertension, refractory hypokalemia, and chloride-resistant metabolic alkalosis due to ectopic adrenocorticotrophic hormone (ACTH) secretion (EAS). In this case report, we discuss the diagnostic challenges of dual paraneoplastic syndromes with SIADH and EAS, management of SCLC with paraneoplastic endocrinopathies, and their prognostic impact on SCLC. In addition, we discuss neuroendocrine differentiation and ectopic hormone production in relation to intratumoral heterogeneity in SCLC and propose tumor microenvironment and hormonal and metabolic dependence as important determinants of tumor growth and survival.

## 1. Introduction

Small-cell lung cancer (SCLC) is an aggressive neuroendocrine subtype of lung cancer and is associated with paraneoplastic syndromes in about 20 to 40% of cases [1, 2]. SIADH and EAS are the most common paraneoplastic endocrinopathies associated with SCLC. The Notch signaling pathway, which mediates cell fate decisions, plays an important role in tumor biology of SCLC. Notch pathway activation inhibits differentiation of SCLC tumor cells into neuroendocrine fate. When the Notch signaling pathway is suppressed, tumor cells remain in neuroendocrine phenotypes and have the potential to secrete various hormones and peptides leading to paraneoplastic syndromes [3, 4].

The association of SCLC with SIADH is well known, with up to 15% of SCLC exhibiting SIADH [5], while 1% to 5% of SCLC has ectopic ACTH secretion resulting in paraneoplastic Cushing syndrome (pCS) [1, 6]. It is very rare to have SCLC with dual ectopic SIADH and ACTH secretion. Only eight cases have been reported in literature [7–14]. Though hyponatremia in SCLC is relatively easy to recognize, EAS can be easily overlooked due to lack of typical Cushingoid picture. Instead, it presents with muscle wasting, weakness, and syndrome of apparent mineralocorticoid excess (SAME), manifesting as resistant hypertension and hypokalemic metabolic alkalosis. In SCLCs with dual SIADH and EAS, the opposing effects of cortisol and ADH on renal sodium excretion can make diagnosis even more challenging. In addition, the presence of these paraneoplastic syndromes is indicative of poor prognosis in SCLC patients, especially EAS carrying the worst prognosis [6]. We present a case of SCLC with hyponatremia at presentation which normalized with the onset of ectopic ACTH secretion.

## 2. Case Description

**Figure 1:**
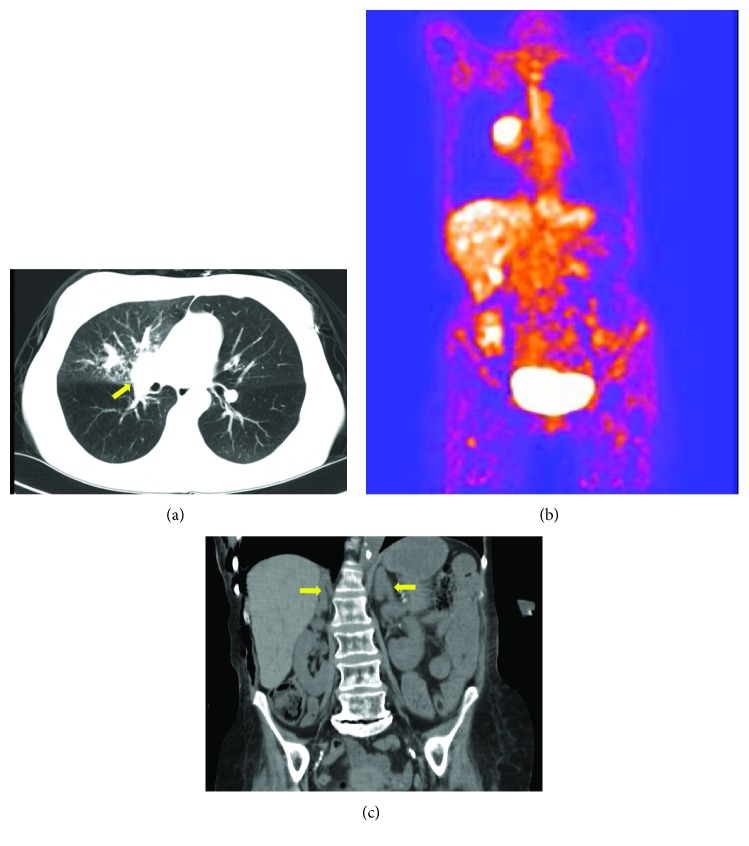
(a) Chest CT (lung window) with a yellow arrow pointing to the right hilar lung primary. (b) PET-CT scan showing an FDG-avid primary tumor in the right lung and metastasis in the liver. (c) Abdomen CT showing bilateral adrenal hypertrophy (yellow arrows).

A 55-year-old female was evaluated for persistent hyponatremia of one-month duration. The physical exam was unremarkable for volume overload or depletion. The workup (Table 1) revealed a sodium level of 126 mmol/l without other electrolyte abnormalities, serum osmolality of 260 mOsm/kg, serum uric acid level of 2.0 mg/dl, normal cortisol, normal TSH, urine sodium of 45 mmol/l, and urine osmolality of 274 mOsm/kg, consistent with SIADH. Citalopram was thought to be the cause of SIADH and stopped. However, persistent hyponatremia prompted a further workup, especially with extensive smoking history and weight loss. Computed tomography showed right hilar mass with metastasis to the liver, right femur, and ribs (Figures 1(a) and 1(b)) with biopsy revealing SCLC.

**Figure 2:**
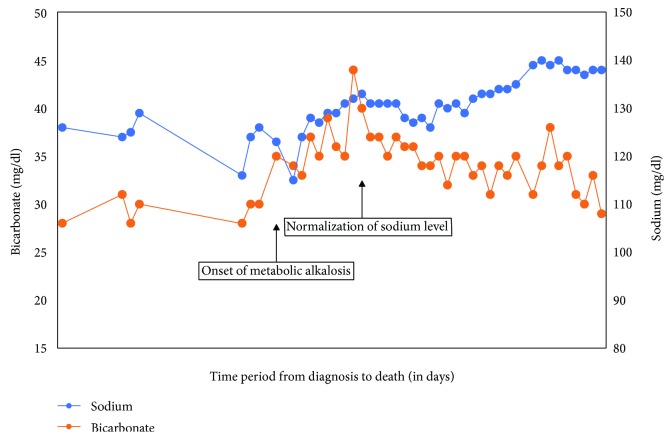
Graph showing serum sodium (blue) and bicarbonate levels (orange) from diagnosis to patient's demise. Note how serum sodium normalizes with onset of metabolic alkalosis.

Despite SCLC diagnosis, the patient continued to smoke cigarettes. Approximately two weeks later, the patient was admitted for acute hypoxic and hypercapnic respiratory failure due to postobstructive pneumonia, COPD exacerbation, and secondary pneumothorax, which were managed with improvement in her respiratory status. However, PaCO_2_ and serum bicarbonate began to increase with the bicarbonate level approaching up to 45 mEq/dl, associated with refractory hypokalemia and uncontrolled hypertension. Metabolic alkalosis was noted to be chloride resistant (urine chloride of >20 mEq/dl). Additionally, hyponatremia which responded moderately to fluid restriction gradually normalized after the onset of metabolic alkalosis (Figure 2). Uncontrolled hypertension, chloride-resistant metabolic alkalosis, and hypokalemia prompted the workup for hyperaldosteronism. Serum aldosterone and plasma renin activity were within normal limits. A high-dose dexamethasone suppression test revealed elevations of ACTH (319 pg/ml) and cortisol (131.5 *μ*g/dl), consistent with ACTH-dependent hypercortisolism and SAME (Table 1) from an ectopic nonsuppressible source of ACTH.

The patient also had significant weight loss of 28 pounds after diagnosis of SCLC, and profound muscle wasting. The second chest CT showed extensive local infiltration of the lung cancer with widespread hepatic metastasis and bilateral adrenal hypertrophy (Figure 1(c)). Palliative chemotherapy was commenced with carboplatin (target AUC—5, dose = 635 mg) and etoposide (100 mg/m^2^ IV). But ACTH and cortisol levels remained elevated (Table 1) despite the first cycle of chemotherapy. Oral ketoconazole (200 mg two times a day) was subsequently started two weeks after chemotherapy. However, the patient did not tolerate the therapy well and continued to deteriorate rapidly with persistent hypercortisolism. Given end-stage disease with poor functional status, palliative care, and comfort measures were pursued as end-of-life care. The patient passed away within 2 months after diagnosis of EAS. Family did not want an autopsy.

## 3. Discussion

We described a case of SCLC with dual sequential paraneoplastic SIADH and EAS in which hyponatremia led to the diagnosis of SCLC, and it gradually faded with the onset of SAME from EAS. SIADH responded moderately to fluid restriction (Na of 126 mmol/l to 130 mmol/l) and the sodium level normalized to about 140 mmol/l after the onset of EAS (Figure 2). Interestingly, EAS may mask SIADH due to the antagonistic action of cortisol and ADH on renal sodium excretion. In EAS, hypercortisolism unmasks the mineralocorticoid action of cortisol due to saturation of 11-betahydroxysteroid dehydrogenase, leading to renal sodium retention. In addition, ADH has been shown to increase plasma ACTH and cortisol levels in patients with Cushing disease, although this relationship is only rarely described in EAS. ACTH response to ADH in EAS appears to depend on vasopressin receptor subtype expression [15, 16].

Hyponatremia is present at presentation in about 15% of patients with SCLC in retrospective studies [17]. However, SIADH is not the only cause of paraneoplastic hyponatremia. Inappropriate secretion of atrial natriuretic peptide (SIANP) has also been documented, and it can also present with hyponatremia due to its pathologic natriuretic effect [18]. Fluid restriction of 1000 ml/day can be applied in SCLC patients newly diagnosed with hyponatremia to differentiate SIADH and SIANP. In our case, hyponatremia responded to fluid restriction, which favored the diagnosis of SIADH rather than SIANP, in which hyponatremia does not respond to 72 to 96 hours of 1000 ml/day fluid restriction and V2 receptor (V2R) antagonists [17].

Management of hyponatremia should be an integral part of SCLC treatment as hyponatremia is associated with a poorer prognosis regardless of an extensive or limited stage. SCLC patients with serum sodium less than 129 mmol/l had a median survival of only 8.63 months compared to 13.6 months in patients with normal sodium, and the degree of hyponatremia is a significant predictor for prognosis [19]. Demeclocycline, which inhibits the effect of ADH in collecting ducts, can be used at a dose of 100 to 300 mg for 3 to 4 times a day. However, its effect is delayed for 1 to 2 weeks. Tolvaptan is also effective in management of SIADH in SCLC patients with sodium level < 125 mmol/l [20]. In our patient, the V2R antagonist was not used as hyponatremia faded with the onset of EAS.

EAS occurs only in 1 to 5% of SCLC patients, and its presentation usually lacks typical Cushingoid features. Patients with EAS usually present with muscle wasting, proximal muscle weakness, and SAME. Diagnosis is established by a high-dose dexamethasone suppression test with nonsuppressible ACTH and cortisol levels. The presence of EAS in SCLC patients confers a very poor prognosis, with a life expectancy of only three to six months [6, 21]. The magnitude of weight loss, rapid decline of performance status, and poor response to chemotherapy makes EAS the most severe of all paraneoplastic syndromes as EAS and SCLC reinforce each other's deleterious effects. The immunosuppression induced by SCLC itself is further amplified by that induced by hypercortisolism, leading to serious infectious complications. Metabolic disorders including steroid-induced hyperglycemia, hypokalemia, and metabolic alkalosis can also significantly worsen general health status [2].

Given its deleterious effects, many authors emphasize treating hypercortisolism before chemotherapy to prevent infectious complications which can be aggravated by cortisol-induced immunosuppression and chemotherapy-induced neutropenia despite the use of granulocyte colony-stimulating factors [1]. Control of severe hypercortisolism before administering chemotherapy may achieve longer survival. Ketoconazole, metyrapone, etomidate, mitotane, and mifepristone can be used to reduce the circulating cortisol level. Ketoconazole was said to have the best tolerance profile [22]. However, being a strong inhibitor of cytochrome P4503A4, ketoconazole may increase the risk of chemotherapy toxicity when used concurrently, and therefore, metyrapone has been reported as a better alternative [23]. For EAS, combinations of metyrapone and ketoconazole or of mitotane, metyrapone, and ketoconazole can be used to control hypercortisolism. If hypercortisolism is refractory to medical therapy, bilateral adrenalectomy might be considered [21, 24].

The occurrence of paraneoplastic syndromes is directly related to the tumor bulk. According to one study, 72% of patients presenting with a paraneoplastic syndrome of any type had extensive disease at diagnosis [25]. EAS is often found when the tumor bulk is particularly large and heterogeneous with three or more organs affected by metastasis [2]. ACTH can be secreted either by the primary tumor or metastatic lesions, a condition referred to as spatial intratumoral heterogeneity. This could reflect an acquisition of new mutations during metastasis and the emergence of clones with secretory ability according to the branched clonal expansion hypothesis [2]. Different SCLC subclones might be able to secrete different ectopic hormones while one clonal type of SCLC cells might also be capable of secreting more than one hormone. Immunohistochemistry of specimens might be helpful to identify origins of ectopic hormones.

Eight cases of SCLC [7–14] with dual SIADH and EAS described in literature are summarized in Table 2. Six [7, 9–11, 13, 14] out of eight cases did not have typical Cushingoid picture and presented with weight loss and SAME. We notice that dual SIADH and EAS in SCLC can be simultaneous or sequential with the latter carrying worse prognosis and shorter life expectancy compared to simultaneous disease. Two reported cases [12, 13] of sequential SIADH and EAS occurred in recurrent disease diagnosed in about 8 months following resolution of the first cancer. Our case is the first reported case of SCLC with sequential SIADH followed by EAS within two months of diagnosis of SCLC with SIADH. All cases of dual sequential SIADH and EAS, including our case, were treatment refractory, and patients died within two months after diagnosis [12, 13].

## 4. Future Implications

Tumor microenvironment and hormonal and metabolic dependence of tumors are important determinants for tumor growth and survival in addition to oncogenic addiction fed by driver gene mutations [26]. SCLC cells can generate their own microenvironment and mediate chemoresistance by transforming a subset of tumor cells into a nonneuroendocrine phenotype via the activation of Notch signaling [3]. One study has shown that endogenous activation of the Notch pathway results in switching from a neuroendocrine to nonneuroendocrine fate in 10 to 50% of tumor cells, generating intratumoral heterogeneity. Nonneuroendocrine Notch-active SCLC cells are slow growing, but they are relatively chemoresistant and provide trophic support to neuroendocrine tumor cells [4, 27]. This finding may provide future implications for Notch status analysis and inhibition in SCLC treatment.

Regarding hormonal dependence, studies have shown that hypercortisolism might induce chemoresistance. In vitro experiments suggested that steroids protect cancer cells from cytotoxic effects of several chemotherapy agents including carboplatin, cisplatin, actinomycin D, and ionizing radiation [28]. This might explain why SCLC with EAS is chemoresistant, as seen in our case. It has been shown that glucocorticoids increase gene expression of several key mediators such as cellular glutathione, metallothionein synthesis, multidrug resistance efflux pump ABCB1 and ABCG2 expression and activity, and O6-methylguanine DNA methyltransferase activity [28, 29]. However, no study has evaluated if SCLC is a hormone-dependent tumor, feeding on its own ectopic hormones. It would be interesting to evaluate if these ectopic hormones, in addition to their target organ effects, also act in autocrine and paracrine fashions on the tumor itself and neighboring cells to influence tumor biology.

Regarding metabolic dependence, we hypothesize that SCLC might have metabolic benefits from hyponatremia and metabolic alkalosis in the tumor microenvironment, which might favor tumor growth and hinder antitumor immune response. The literature mostly describes the prevalence of hyponatremia in SCLC patients along with its poor prognosis. However, there is no study yet to evaluate the effect of hyponatremia on tumor growth, chemoresistance, and antitumor immune response. In the hyponatremic milieu, cells may leak out organic osmoles including glycine, glutamate, and inositol to maintain osmotic balance with extracellular fluid [30]. We hypothesize that hyponatremia might promote tumor growth by providing inositol and glutamate from neighboring cells in tumor microenvironment, and it might also impair cytotoxic activity of CD8+ T lymphocytes and NK cells via SGK signaling [31]. According to one recent study, glutamate inhibits the xCT glutamate-cystine antiporter, leading to intracellular cysteine depletion. EglN1, the main HIF1 prolyl hydroxylase, undergoes oxidative self-inactivation in the absence of cysteine, resulting in HIF1 accumulation and cellular proliferation via the pseudohypoxic pathway [32]. Further studies are needed to shed light into the effects of paraneoplastic endocrinopathies and their resultant metabolic effects on the tumor growth, metastasis, and immune response.

## Figures and Tables

**Table 1:** Some important laboratory results. D stands for day. ^∗^Patient passed away on D78.

Test	Initial office visit (D0)	After the onset of EAS (D47)	Chemotherapy day 1 of 3 (D53)	Postchemo day 8 (D63)	MICU admission (D72)	Comfort measures initiated (D77) ^∗^
Hemoglobin (g/dl)	12.7	12.9	8.6	8.5	8.2	6.7
Hematocrit (%)	35.1	37.9	25.4	24.4	24.3	20.1
WBC (10^3^/mm^3^)	7.4	19.4	14.7	0.3	20.6	16.3
Platelet count (10^3^/mm^3^)	255	200	168	23	366	379
Sodium (mEq/l)	126	135	128	134	139	138
Potassium (mEq/l)	4.3	2.8	4.4	3.3	4.1	5.3
Chloride (mEq/l)	83	76	84	87	92	97
Bicarbonate (mEq/l)	28	45	34	34	37	29
Anion gap (mEq/l)	15	14	10	13	10	12
Glucose (mg/dl)	82	98	111	118	127	131
BUN (mg/dl)	11	11	27	9	11	10
Creatinine (mg/dl)	0.6	0.5	0.6	0.5	0.5	0.5
S. osm (mOsm/kg)	260					
Calcium (mEq/l)	9	9				Ical—1.27
AST (U/l)	25			17		14
ALT (U/l)	18			33		20
T. bilirubin (mg/dl)	0.3			0.4		0.1
Troponin (ng/ml)	0.01					
ACTH (pg/ml)		319	265	399		
Cortisol (*μ*g/dl)	7.1	131.5		164.4	138.5	134.7
TSH (mIU/l)	0.88					
PRA		<0.15				
Aldosterone (ng/dl)		<1.0				
Epinephrine (pg/ml)					47	
Norepinephrine (pg/ml)					1004	
Dopamine (pg/ml)					126	
DHEA sulfate (*μ*g/dl)			60			

**Table 2:** Summary of previously published case reports of SCLC with dual ectopic ADH and ACTH secretion.

Liddle et al.	SCLC with SIADH and EAS. Chronology of SIADH and EAS was not mentioned. Na was 115 mmol/l. Clinical picture of EAS, management, and life expectancy were not described [7].
O'Neal et al.	Extensive SCLC with >3 organ metastases (liver, adrenals, brain, diaphragm, and retroperitoneal and mediastinal lymph nodes) and simultaneous SIADH and EAS, presenting with uncontrolled hypertension, puffy face, and hyponatremia (Na 121–125 mmol/l). The patient had a gradual development of hypokalemic metabolic alkalosis and Cushingoid picture in 2 months and died after 5 weeks of diagnosis of dual SIADH and EAS. Management was not described [8].
Coscia et al.	Extensive SCLC (3 cm in size) with >3 organ metastasis (adrenals, pancreas, mediastinal lymph nodes, bone marrow, liver, and spleen) and simultaneous SIADH and EAS presenting with symptomatic, profound hyponatremia (Na 103 mmol/l), hypertension, hemoptysis, and weight loss of 10 pounds without typical Cushingoid picture. The patient also had few weeks of nausea, vomiting, anorexia, and diarrhea before presentation. Hyponatremia was treated with 3% saline and fluid restriction. No antisteroid agent was used for EAS. The patient died on the 24th hospital day [9].
Suzuki et al.	Extensive SCLC with >3 organ metastasis (adrenals, contralateral lung, pleurae, liver, bone marrow, pancreas, spleen, thyroid, and multiple lymph nodes) and simultaneous SIADH and EAS presenting with significant weight loss in 6 weeks, cough, dyspnea, uncontrolled hypertension, hyperglycemia, muscle weakness, hypokalemic metabolic alkalosis, and hyponatremia (Na 126 mmol/l). The patient did not have typical Cushingoid picture. Treatment of SIADH and EAS was not mentioned. The patient was treated with nimustine without success. The patient died from severe pancytopenia and GI bleeding on the 42nd hospital day [10].
Pierce et al.	Extensive SCLC (2 cm in size) with 3 organ metastases (right adrenal gland, thyroid, and pancreas) and simultaneous SIADH and EAS, presenting with hyponatremia (Na 126 mmol/l), hypokalemia, metabolic alkalosis, hyperglycemia, hypertension, and 15-pound weight loss in one month. No typical Cushingoid picture was identified. SCLC was treated with cisplatin and etoposide; SIADH was treated with fluid restriction and demeclocycline; EAS was treated with aminoglutethimide. Despite all treatments, ADH and ACTH levels remained elevated. The patient died 127 days after diagnosis [11].
Shaker et al.	Metastatic extrapulmonary small-cell carcinoma in the bone marrow presenting with renal phosphate wasting and SIADH (Na 107 mmol/l). SIADH responded to fluid restriction and demeclocycline. There was a resolution of cancer with adriamycin, cyclophosphamide, cisplatin, and etoposide 5 months after diagnosis. Eight months after diagnosis, patient presented with bone pain, adenopathy, Cushingoid picture, hypokalemia, hypertension, and recurrent SIADH. No antisteroid agent was used for EAS. The patient died 2 months after the onset of EAS and SIADH, despite chemotherapy [12].
Mayer et al.	SCLC (3.5 cm in size) with dual sequential SIADH and EAS, initially presenting with limited-stage SCLC diagnosed from SIADH workup (Na 123 mmol/l). SIADH responded to fluid restriction, and SCLC achieved a complete remission after 4 cycles of carboplatin, etoposide, and concurrent radiation therapy. EAS occurred 8 months after diagnosis, presenting with weight loss of 20 pounds in 2 weeks, hypokalemia, muscle weakness, hyperglycemia, and hypertension. The patient had metastatic disease in the liver, pericardial lymph nodes, bilateral adrenal glands, and the mesenteric fat. The patient died 2 days after diagnosis of EAS [13].
Müssig et al.	Extensive SCLC (4.9 cm × 10.6 cm in size) with 2 organ metastases (liver and brain) and simultaneous SIADH and EAS, presenting with 28-pound weight loss over 6 months, persistent hypokalemia, and hyponatremia (Na of 116 mmol/l). EAS lacked typical Cushingoid picture. SIADH was treated with fluid restriction only. No antisteroid agent was used for EAS. A nearly complete radiological remission with resolution of SIADH and EAS was achieved after the fourth cycle of carboplatin and etoposide. Life expectancy was not mentioned [14].

## References

[1] Shepherd F. A., Laskey J., Evans W. K., Goss P. E., Johansen E., Khamsi F. (1992). Cushing's syndrome associated with ectopic corticotropin production and small-cell lung cancer. *Journal of Clinical Oncology*.

[2] Nagy-Mignotte H., Shestaeva O., Vignoud L. (2014). Prognostic impact of paraneoplastic Cushing’s syndrome in small-cell lung cancer. *Journal of Thoracic Oncology*.

[3] Ito T., Kudoh S., Ichimura T., Fujino K., Hassan W. A. M. A., Udaka N. (2017). Small cell lung cancer, an epithelial to mesenchymal transition (EMT)-like cancer: significance of inactive Notch signaling and expression of achaete-scute complex homologue 1. *Human Cell*.

[4] Lim J. S., Ibaseta A., Fischer M. M. (2017). Intratumoural heterogeneity generated by Notch signalling promotes small-cell lung cancer. *Nature*.

[5] Hansen O., Sørensen P., Hansen K. H. (2010). The occurrence of hyponatremia in SCLC and the influence on prognosis. *Lung Cancer*.

[6] Delisle L., Boyer M. J., Warr D. (1993). Ectopic corticotropin syndrome and small-cell carcinoma of the lung. *Archives of Internal Medicine*.

[7] Liddle G. W., Givens J. R., Nicholson W. E., Island D. P. (1965). The ectopic ACTH syndrome. *Cancer Research*.

[8] O'Neal L. W., Kipnis D. M., Luse S. A., Lacy P. E., Jarett L. (1968). Secretion of various endocrine substances by ACTH-secreting tumors—gastrin, melanotropin, norepinephrine, serotonin, parathormone, vasopressin, glucagon. *Cancer*.

[9] Coscia M., Brown R. D., Miller M. (1977). Ectopic production of antidiuretic hormone (ADH), adrenocorticotrophic hormone (ACTH) and beta-melanocyte stimulating hormone (*β*-MSH) by an oat cell carcinoma of the lung. *The American Journal of Medicine*.

[10] Suzuki H., Tsutsumi Y., Yamaguchi K., Abe K., Yokoyama T. (1984). Small cell lung carcinoma with ectopic adrenocorticotropic hormone and antidiuretic hormone syndromes: a case report. *Japanese Journal of Clinical Oncology*.

[11] Pierce S. T., Metcalfe M., Banks E. R., O'Daniel M. E., Desimone P. (1992). Small cell carcinoma with two paraendocrine syndromes. *Cancer*.

[12] Shaker J. L., Brickner R. C., Divgi A. B., Raff H., Findling J. W. (1995). Case report: renal phosphate wasting, syndrome of inappropriate antidiuretic hormone, and ectopic corticotropin production in small cell carcinoma. *The American Journal of the Medical Sciences*.

[13] Mayer S., Cypess A. M., Kocher O. N. (2005). Uncommon presentations of some common malignancies. *Journal of Clinical Oncology*.

[14] Müssig K., Horger M., Häring H. U., Wehrmann M. (2007). Syndrome of inappropriate antidiuretic hormone secretion and ectopic ACTH production in small cell lung carcinoma. *Lung Cancer*.

[15] Colombo P., Passini E., Re T., Faglia G., Ambrosi B. (1997). Effect of desmopressin on ACTH and cortisol secretion in states of ACTH excess. *Clinical Endocrinology*.

[16] Arlt W., Dahia P. L. M., Callies F. (1997). Ectopic ACTH production by a bronchial carcinoid tumour responsive to desmopressin in vivo and in vitro. *Clinical Endocrinology*.

[17] Chute J. P. (2006). A metabolic study of patients with lung cancer and hyponatremia of malignancy. *Clinical Cancer Research*.

[18] Sun N. H., Wang S. H., Liu J. N. (2017). The productions of atrial natriuretic peptide and arginine vasopressin in small cell lung cancer with brain metastases and their associations with hyponatremia. *European Review for Medical and Pharmacological Sciences*.

[19] Wang W., Song Z., Zhang Y. (2016). Hyponatremia in small cell lung cancer is associated with a poorer prognosis. *Translational Cancer Research*.

[20] Petereit C., Zaba O., Teber I., Lüders H., Grohé C. (2013). A rapid and efficient way to manage hyponatremia in patients with SIADH and small cell lung cancer: treatment with tolvaptan. *BMC Pulmonary Medicine*.

[21] Zhang H., Zhao J. (2017). Ectopic Cushing syndrome in small cell lung cancer: a case report and literature review. *Thoracic Cancer*.

[22] Tabarin A., Navarranne A., Guérin J., Corcuff J. B., Parneix M., Roger P. (1991). Use of ketoconazole in the treatment of Cushing's disease and ectopic ACTH syndrome. *Clinical Endocrinology*.

[23] Aziz S. I., Khattak M. A., Usmani Z., Ladipeerla N., Pittman K. (2011). Metyrapone: a management option for ectopic ACTH syndrome in small cell lung cancer treated with intravenous etoposide. *Case Reports*.

[24] Kanaji N., Watanabe N., Kita N. (2014). Paraneoplastic syndromes associated with lung cancer. *World Journal of Clinical Oncology*.

[25] Gandhi L., Johnson B. E. (2006). Paraneoplastic syndromes associated with small cell lung cancer. *Journal of the National Comprehensive Cancer Network*.

[26] Luo J., Solimini N. L., Elledge S. J. (2009). Principles of cancer therapy: oncogene and non-oncogene addiction. *Cell*.

[27] Hassan W. A., Yoshida R., Kudoh S. (2016). Notch1 controls cell chemoresistance in small cell lung carcinoma cells. *Thoracic Cancer*.

[28] Mitre-Aguilar I., Cabrera-Quintero A., Zentella-Dehesa A. (2015). Genomic and non-genomic effects of glucocorticoids: implications for breast cancer. *International Journal of Clinical and Experimental Pathology*.

[29] Rutz H. P. (2002). Effects of corticosteroid use on treatment of solid tumours. *The Lancet*.

[30] Mcmanus M. L., Churchwell K. B., Strange K. (1995). Regulation of cell volume in health and disease. *New England Journal of Medicine*.

[31] Shi G., Wang Q., Zhou X. (2017). Response of human non-small-cell lung cancer cells to the influence of Wogonin with SGK1 dynamics. *Acta Biochimica et Biophysica Sinica*.

[32] Briggs K. J., Koivunen P., Cao S. (2016). Paracrine induction of HIF by glutamate in breast cancer: EglN1 senses cysteine. *Cell*.

